# Heterologous expression of cytotoxic sesquiterpenoids from the medicinal mushroom *Lignosus rhinocerotis* in yeast

**DOI:** 10.1186/s12934-017-0713-x

**Published:** 2017-06-12

**Authors:** Hui-Yeng Yeannie Yap, Mariano Jordi Muria-Gonzalez, Boon-Hong Kong, Keith A. Stubbs, Chon-Seng Tan, Szu-Ting Ng, Nget-Hong Tan, Peter S. Solomon, Shin-Yee Fung, Yit-Heng Chooi

**Affiliations:** 1 Department of Molecular Medicine, Faculty of Medicine, University of Malaya, 50603 Kuala Lumpur, Malaysia; 2 Research School of Biology, The Australian National University, Canberra, Australia; 3 School of Molecular Sciences, University of Western Australia, Crawley, WA 6009 Australia; 4 Ligno Biotech, 43300 Balakong Jaya, Selangor Malaysia; 5 Centre for Crop and Disease Management, Curtin University, Perth, WA 6102 Australia

**Keywords:** *Lignosus rhinocerotis*, Tiger milk mushroom, Sesquiterpene synthase, Sesquiterpenoid, (+)-Torreyol, α-Cadinol, Heterologous expression, *Saccharomyces cerevisiae*

## Abstract

**Background:**

Genome mining facilitated by heterologous systems is an emerging approach to access the chemical diversity encoded in basidiomycete genomes. In this study, three sesquiterpene synthase genes, GME3634, GME3638, and GME9210, which were highly expressed in the sclerotium of the medicinal mushroom *Lignosus rhinocerotis*, were cloned and heterologously expressed in a yeast system.

**Results:**

Metabolite profile analysis of the yeast culture extracts by GC–MS showed the production of several sesquiterpene alcohols (C_15_H_26_O), including cadinols and germacrene D-4-ol as major products. Other detected sesquiterpenes include selina-6-en-4-ol, β-elemene, β-cubebene, and cedrene. Two purified major compounds namely (+)-torreyol and α-cadinol synthesised by GME3638 and GME3634 respectively, are stereoisomers and their chemical structures were confirmed by ^1^H and ^13^C NMR. Phylogenetic analysis revealed that GME3638 and GME3634 are a pair of orthologues, and are grouped together with terpene synthases that synthesise cadinenes and related sesquiterpenes. (+)-Torreyol and α-cadinol were tested against a panel of human cancer cell lines and the latter was found to exhibit selective potent cytotoxicity in breast adenocarcinoma cells (MCF7) with IC_50_ value of 3.5 ± 0.58 μg/ml while α-cadinol is less active (IC_50_ = 18.0 ± 3.27 μg/ml).

**Conclusions:**

This demonstrates that yeast-based genome mining, guided by transcriptomics, is a promising approach for uncovering bioactive compounds from medicinal mushrooms.

**Electronic supplementary material:**

The online version of this article (doi:10.1186/s12934-017-0713-x) contains supplementary material, which is available to authorized users.

## Background

Basidiomycete mushrooms are important sources of bioactive secondary metabolites including terpenoids, alkaloids, and polyketides [1]. Exploration of the medicinal and pharmacological potential of these underexplored mushroom species could be scientifically and biotechnologically rewarding. *Lignosus rhinocerotis* (Cooke) Ryvarden (orthographic variant is *L. rhinocerus*; common name: tiger milk mushroom) belongs to the order Polyporales (Polyporaceae family). It has been found in tropical regions in China, East Africa, Sri Lanka, Thailand, Philippines, Indonesia, Papua New Guinea, Australia, Vanuatu, and Malaysia [2–4]. Lau et al. [5] has compiled the ethnomycological uses of the sclerotia of the mushroom by different communities including the Malay, Chinese, and several sub-tribes of indigenous communities in Peninsular Malaysia including the Temuans and Semai aborigines to treat coughs, asthma, tuberculosis, and cancer; where several preparation methods of the fungus for medicinal purposes have been documented. Both aqueous (e.g., decoction) and non-aqueous preparations (e.g., tincture) have been reported, which may indicate the presence of hydrophilic and lipophilic active substances, respectively [5].

The genome and transcriptome studies of *L. rhinocerotis* have provided insights into the biology of this medicinal mushroom and opened up new opportunities for exploration via genomic approaches [6–9]. Transcriptomics analysis revealed genes encoding several small secreted cysteine-rich proteins (cerato-platanins and hydrophobins) and lectins were among the highest expressed genes in the sclerotium of *L. rhinocerotis* and may have important implications for various bioactivities relevant to human diseases [7]. On the other hand, the *L. rhinocerotis* genome harbours genes encoding a number of secondary metabolite biosynthetic enzymes, including 12 sesquiterpene synthases (STSs), one non-ribosomal peptide synthetase (NRPS), and a polyketide synthase (PKS) [6]. These enzymes are crucial for the biosynthesis of sesquiterpenes, non-ribosomal peptides, and polyketides, respectively. Among them, six secondary metabolite gene clusters harbouring genes encoding four STS genes and one each of NRPS and PKS were also found to express at notable levels in the *L. rhinocerotis* sclerotium [7].

Genome mining has emerged as a potential avenue to access the chemical diversity encoded in basidiomycete fungal genomes [10]. The large number of STS genes in the genome highlights the potential of *L. rhinocerotis* in producing diverse sesquiterpenoids. Many sesquiterpenoids have potent antibiotic and cytotoxic activities due to their high chemical reactivity [11]. Some renowned fungal sesquiterpenoids include the anticancer active illudins from *Omphalotus olearius* [12], the cytotoxic PR toxin (from *Penicillium roqueforti*) with robust antimicrobial activity [13], and the platelet aggregation inhibitor radulone A from *Radulomyces confluens* [11]. The basidiomycete STSs have been shown to be readily expressed in well-established laboratory hosts, such as *Escherichia coli* and *Saccharomyces cerevisiae* [12, 14]. This makes heterologous expression an attractive approach for accessing the basidiomycete terpenoid chemistry, as many basidiomycete fungi are relatively slow growing in the laboratory environment.

We have only begun to appreciate the diversity of STSs in basidiomycetes [10]. Nonetheless, sesquiterpene biosynthesis of Polyporales which consist of several medicinal mushrooms including *Ganoderma lucidum* [15], *Antrodia cinnamomea* [16], *Trametes versicolor* [17] and *L. rhinocerotis* (this study), remained largely unexplored. Given that *S. cerevisiae* has proven to be extremely useful for genome mining and characterisation of fungal secondary metabolite pathways [18] as well as for commercial production of terpenoids [19], we have chosen to use the yeast as an expression host for *L. rhinocerotis* STSs in this study. Guided by previous transcriptomic data, we cloned several sclerotium-expressed *L. rhinocerotis* STS genes for heterologous expression in a *S. cerevisiae* yeast system. The putative products from these STSs were identified by gas chromatography-mass spectrometry (GC–MS). Two major sesquiterpene alcohol products were isolated and further characterised structurally. The potential cytotoxic activities of the two compounds against a panel of human cancer cell lines were also examined.

## Methods

### Mushroom material and DNA extraction

*L. rhinocerotis* TM02 strain (TM02) was obtained from LiGNO™ Biotech (Selangor, Malaysia). The fungus was authenticated by its nuclear ribosomal internal transcribed spacer (ITS) region using FTM and ITS4 primers [20]. A voucher specimen was deposited at Royal Botanic Gardens, Kew (London, UK) with the accession number K(M) 177812. DNA was extracted using a modified hexadecyl trimethyl-ammonium bromide method [21].

### Gene cloning and plasmid construction

The coding sequences of the three STS genes, *GME3634*, *GME3638*, and *GME9210*, in the sequenced TM02 genome, have been revised based on the recent RNA sequencing data [6, 7]. The revised coding sequences are presented in Additional file 1: Table S1 and deposited in GenBank for public access under accession numbers KX281943, KX281944, and KX281945, respectively. Individual exons of the STS genes were amplified by PCR with 35–40 bp overhangs that overlap with neighbouring exons or plasmid backbone and stitched together to form the respective ‘intronless’ cDNA genes by yeast-mediated in vivo DNA recombination. The oligonucleotide primers used for constructing the genes are listed in Additional file 1: Table S2. PCR reactions were performed using Q5^®^ High-Fidelity DNA Polymerase (New England Biolabs) according to the manufacturer’s protocol at an annealing temperature of 66 °C. In vivo yeast recombination cloning was carried out using a Frozen-EZ Yeast Transformation II Kit™ (Zymo Research) where competent *S. cerevisiae* BJ5464 [22] was transformed with the DNA fragments (PCR products) of the respective genes and linearized plasmid backbone derived from YEplac-ADH2p [23, 24]. Yeast transformants grown on synthetic dropout agar plate lacking uracil were screened by PCR. Plasmid DNA was isolated from positive transformants using a Zymoprep™ Yeast Plasmid Miniprep II Kit (Zymo Research) and used to transform NEB 5-alpha Electrocompetent *E. coli* (New England Biolabs) by electroporation. Plasmid DNA was isolated from the positive clones grown on LB-ampicillin using the alkaline lysis method and further verified by DNA sequencing.

### Heterologous expression in yeast system

*Saccharomyces cerevisiae* BJ5464 was transformed with the three verified STSs (*GME3634*, *GME3638*, or *GME9210*) expression constructs. For small scale analysis, individual single yeast colonies were grown in 3 ml of yeast synthetic dropout medium lacking uracil as a starter culture for 72 h at 28 °C under shaking conditions (200 rpm). A total of 100 ml YPD (or yeast extract-peptone-dextrose) broth was inoculated with 100 µl of the starter culture and incubated for 90 h at 28 °C under shaking conditions (200 rpm). Cells were pelleted at 5000×*g* for 15 min and extracted with an equal volume of acetone at room temperature (RT). Samples were analysed by GC–MS as described below. For large scale production of the sesquiterpenes, the *S. cerevisiae* BJ5464 harbouring *GME3634*, *GME3638*, or *GME9210*, were grown in 7 ml of synthetic dropout medium lacking uracil as a starter culture for 72 h at 28 °C under shaking conditions (200 rpm). Six millilitres of the starter culture were inoculated into 6 l of YPD broth and incubated 90 h at 28 °C under shaking conditions (200 rpm). Cells were pelleted as before and extracted with acetone.

### Isolation, purification, and identification of sesquiterpenes

The extraction of sesquiterpenes from yeast was performed as described previously [25]. The yeast cell pellet was extracted with an equal volume of acetone twice at room temperature and concentrated by rotary evaporator to remove the acetone from the extract. The aqueous portion was partitioned twice with dichloromethane (DCM). The DCM fraction was then subjected to silica gel (SiO_2_) flash chromatography separation with a simple linear 100% hexane to 100% ethyl acetate gradient using a Reveleris^®^ X2 Flash Chromatography System (GRACE, Columbia, MD, USA). Fractions were analysed by GC–MS on a Shimadzu GC–MS QP2010 to confirm the presence of the sesquiterpene. Samples were injected into the injector port at 250 °C in splitless mode and eluted with helium into a Rtx-5MS column (30.0 m length × 0.25 mm inner diameter × 0.25 µm film thickness) (Restek, Bellefonte, PA, USA). The following GC oven temperature program was applied: 35 °C hold for a minute, 30 °C/min ramp to 70 °C and hold for 2 min, followed by a 15 °C/min ramp to 159 °C, then a 6 °C/min ramp to 217 °C and finally a 16 °C/min ramp to 325 °C and hold for 3 min (total program time: 29.52 min). The software used for the acquisition and data analysis was GCMSsolution software (Shimadzu, Kyoto, Japan). Compounds were putatively identified by comparing the mass spectra (45–320 m/z range) to the NIST 05 library (NIST, Gaithersburg, MD, USA). ^1^H, ^13^C, and 2D NMR spectra of (+)-torreyol and α-cadinol were obtained in deuterated chloroform (CDCl_3_) using either a Bruker Avance IIIHD 500 or 600 MHz spectrometer. The optical rotations of (+)-torreyol and α-cadinol were determined using an Autopol 1 polarimeter (Rudolph Research Analytical, Hackettstown, NJ, USA).

### Phylogenetic tree construction

Close protein sequence homologs of GME3634, GME3638, and GME9210 obtained from NCBI using BLASTP (E value <1e-114, Identity >47%), along with several previously reported and characterised STSs from *Coprinus cinereus* (Cop1, Cop2, Cop3, Cop4, Cop5, Cop6) [14] and *O. olearius* (Omp1, Omp2, Omp3, Omp4, Omp5a, Omp5b, Omp6, Omp7, Omp8. Omp9, Omp10) [12], were used for phylogenetic analysis. In addition, protein sequences of two STSs from *Arabidopsis thaliana*, At5g44630 and At5g23960 [26], were included to root the phylogenetic tree. The protein sequences were aligned with the sequence alignment integrated tool (ClustalW) from MEGA6 [27] and the poorly alignable regions were removed by the GBlocks server using a less stringent selection [28]. The ProtTest 2.4 server was used to select the best fit empirical substitution model of protein evolution based on the Bayesian information criterion [29]. A bootstrap test of phylogeny to construct maximum-likelihood tree was conducted using MEGA6 with 1000 replicates based on the Le_Gascuel_2008 (LG) model [30]. From this, a single consensus tree with ≥ 70% majority-rule was built.

### Cell viability assay

The 3-(4,5-dimethylthiazol-2-yl)-2,5-diphenyltetrazolium bromide (MTT) colorimetric assay was used to assess cell viability where the yellow tetrazolium dye is reduced to the insoluble, purple crystalline formazan in metabolically active viable cells by NAD(P)H-dependent cellular oxidoreductases. Mammary gland epithelium (184B5, CRL-8799™), epithelial mammary gland adenocarcinoma (MCF7, HTB-22™), epithelial lung carcinoma (A549, CCL-185™), and epithelial prostate adenocarcinoma (PC-3, CRL-1435™) cells were purchased from ATCC^®^ (Manassas, VA). MCF7, A549, and PC-3 cell lines were maintained in Roswell Park Memorial Institute (RPMI)-1640 while 184B5 cell line was cultured in Mammary Epithelial Cell Growth Medium (MEGM) bullet kit. Monolayer cells grown in 96-well plate were treated with the isolated sesquiterpenes dissolved in ≤0.4% dimethyl sulfoxide (DMSO) at different concentrations (0.625–40 µg/ml). The MTT solution of 5 mg/ml in phosphate-buffered saline solution (PBS) was added 72 h post-treatment and the plate was incubated at 37 °C for an additional 4 h until formazan developed. The MTT-containing spent medium was aspirated out and an equal amount of DMSO was added to dissolve the formazan and absorption was measured at 570 nm. The IC_50_ value was determined from the cell viability percentages against the concentrations curve. The untreated and vehicle-treated cells served as control groups.

## Results

### Heterologous expression of *L. rhinocerotis* STS genes resulted in the production of diverse sesquiterpenes in yeast

**Fig. 1:**
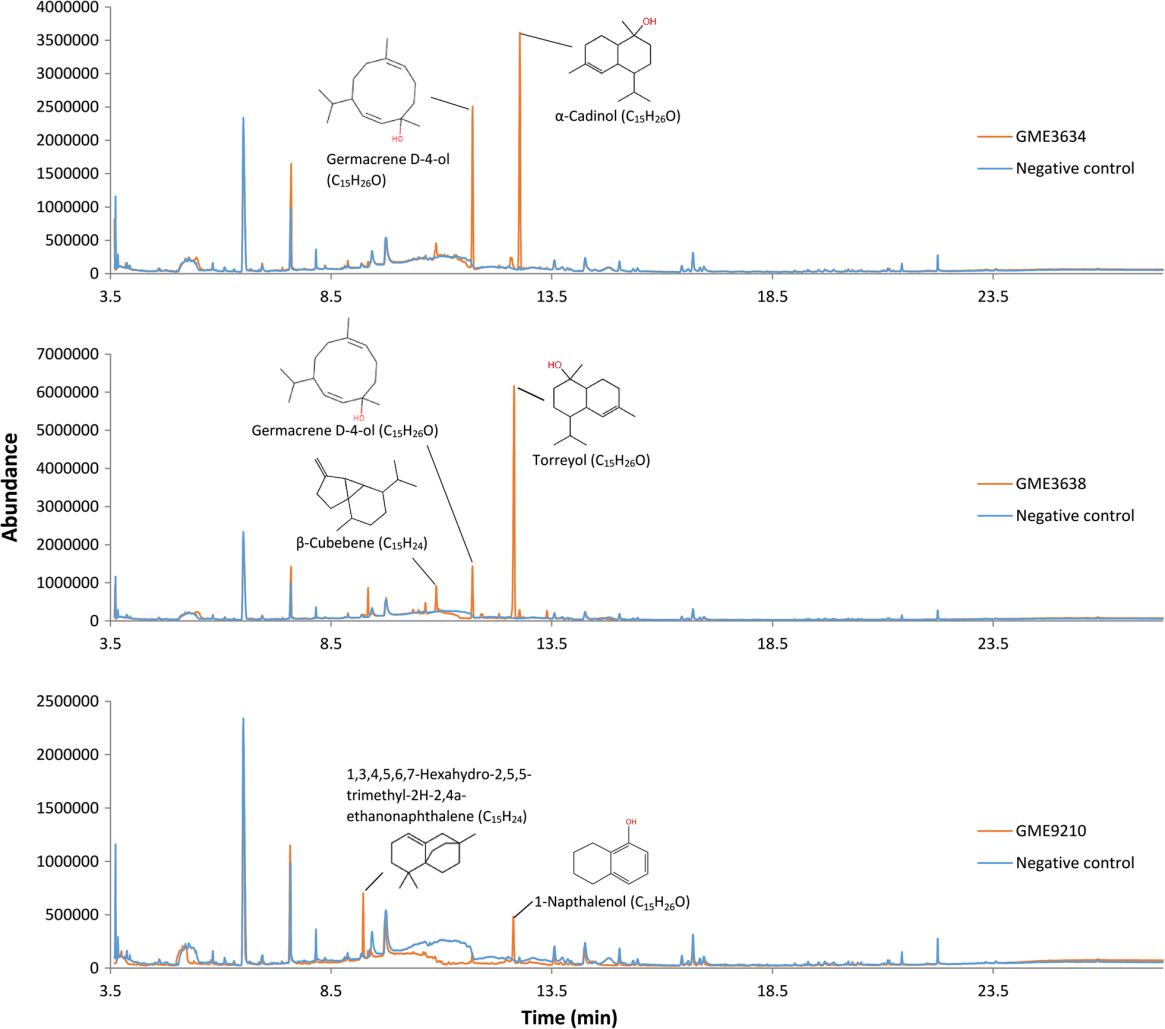
Heterologous production of sesquiterpenes in *S. cerevisiae* BJ5464 expressing GME3634, GME3638, and GME9210. GC–MS profile of the culture extracts in comparison to the profile of the empty vector (control)

Genome sequencing revealed that *L. rhinocerotis* is rich in STS genes (12 in total) [6] while transcriptome study showed that seven out of the 12 terpene synthase genes are actively expressed in the sclerotium, which is the part of the mushroom used in traditional medicine [7]. Based on this reported data from previous literature, we attempted to express the three terpene synthase genes *GME3634*, *GME3638*, and *GME9210* that were most highly expressed in the sclerotium. *GME3634* and *GME3638* clustered together on scaffold 22 which also includes a gene encoding an aldo/keto reductase (*GME3639*) while *GME9210* forms another terpene gene cluster together with a NAD-P-binding protein and an ABC transporter located at scaffold 346 of the *L. rhinocerotis* genome assembly [6, 7]. Comparison of the GC–MS profiles of the yeast cultures expressing GME3634, GME3638, or GME9210 against the empty vector yeast control revealed the putative sesquiterpene alcohols (C_15_H_26_O) germacrene D-4-ol and cadinols as the major products. Other minor compounds were putatively identified as selina-6-en-4-ol, β-elemene, β-cubebene, and cedrene based on strong matches with spectra in the NIST database (Table 1). Among the synthases, GME3638 produced the highest number of sesquiterpenes, 19 in total and six of them were sesquiterpene alcohols, followed by GME3634 which produced eight sesquiterpenes including five alcohols, and GME9210 with two sesquiterpenes, one of them an alcohol. No sesquiterpenes were detected in the empty vector control. GC–MS metabolite profiles of the culture extracts are shown in Fig. 1.

**Table 1:** Heterologous production of sesquiterpenes in *S. cerevisiae* BJ5464 expressing GME3634, GME3638, and GME9210

RT (min)	SI (%)	Peak area	Formula	Name	Structure
GME3634					
10.315	65	400,382	(C_15_H_24_)	Unknown sesquiterpene	
10.790	74	282,208	(C_15_H_24_)	Unknown sesquiterpene	
10.855	68	187,589	(C_15_H_24_)	Unknown sesquiterpene	
11.370	91	3,367,644	C_15_H_26_O	Germacrene D-4-ol	
11.820	75	66,158	(C_15_H_26_O)	Unknown sesquiterpene	
12.035	89	525,206	C_15_H_26_O	τ-Muurolol	
12.080	74	84,666	(C_15_H_26_O)	Unknown sesquiterpene	
12.175	95	4,408,840	C_15_H_26_O	α-Cadinol	
GME3638					
9.510	92	501,063	C_15_H_24_	α-Copaene	
9.590	88	506,236	C_15_H_24_	β-Elemene	
9.655	94	4,396,543	C_15_H_24_	β-Elemene	
9.915	76	197,793	(C_15_H_24_)	Unknown sesquiterpene	
10.200	86	764,201	C_15_H_24_	Isoledene	
10.315	55	707,826	(C_15_H_24_)	Unknown sesquiterpene	
10.430	89	1,820,157	C_15_H_24_	Germacrene D	
10.480	94	1,041,092	C_15_H_24_	Germacrene D	
10.590	87	1,068,119	C_15_H_24_	β-Cubebene	
10.645	95	2,408,088	C_15_H_24_	α-Muurolene	
10.855	83	1,246,428	C_15_H_24_	δ-Cadinene	
10.940	68	603,336	(C_15_H_24_)	Unknown sesquiterpene	
11.455	69	254,072	(C_15_H_24_)	Unknown sesquiterpene	
10.795	82	3,768,107	C_15_H_26_O	Selina-6-en-4-ol	
11.375	94	8,944,970	C_15_H_26_O	Germacrene D-4-ol	
11.510	75	963,361	(C_15_H_26_O)	Unknown sesquiterpene	
11.830	86	805,039	C_15_H_26_O	Germacrene D-4-ol	
12.100	95	41,559,921	C_15_H_26_O	Torreyol	
12.180	93	1,713,657	C_15_H_26_O	α-Cadinol	
GME9210					
9.535	88	734,814	C_15_H_24_	1,3,4,5,6,7-Hexahydro-2,5,5-trimethyl-2H-2,4a-ethanonaphthalene	
11.375	74	512,708	(C_15_H_26_O)	Unknown sesquiterpene	

Possible candidate with the highest SI is shown for compound identification. Sesquiterpenes with SI lower than 80 but with predicted molecular formula typical of sesquiterpenes are listed as unknown sesquiterpene

*RT* retention time, *SI* similarity index

A single peak with a retention time (RT) of 12.1 min identified as torreyol (syn. δ-cadinol) with significant abundance was consistently present in the profile of GME3638-expressing yeast. GME3634 produced two notable peaks with moderate abundances at RT of 11.37 and 12.18 min identified as germacrene D-4-ol and α-cadinol respectively. In contrast, GME9210 only produced sesquiterpenes at comparatively low abundances, including 1,3,4,5,6,7-hexahydro-2,5,5-trimethyl-2H-2,4a-ethanonaphthalene (Fig. 1). Therefore, we focused on isolating and identifying the major compounds from GME3638 and GME3634.

### GME3638 and GME3634 expression in yeast produced (+)-torreyol and α-cadinol

**Fig. 2:**
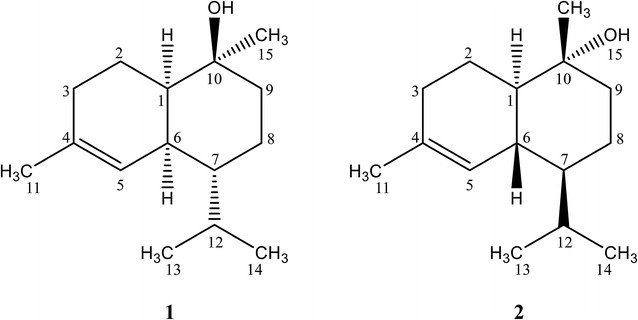
Structures of (+)-torreyol (*1*) and α-cadinol (*2*)

Extraction from six litre cultures of GME3638- and GME6364-expressing yeast enabled the purification of 5.1 ± 0.50 and 1.0 ± 0.25 mg of the two putative compounds namely torreyol (RT = 12.1 min) and α-cadinol (RT = 12.18 min) (Fig. 1). Mass spectra of the isolated compounds compared to those compounds in the databases are shown in Additional file 1: Figure S1. Both compounds presented a similarity index of 95% in the NIST library, which is highly significant. NMR experiments were performed to confirm the identities of the compounds with the compound produced by GME3638 corresponding to (+)-torreyol, while GME3634 produced α-cadinol (Additional file 1: Table S3; Fig. 2).

Polarimetric analysis showed that the optical rotation of both compounds corresponded to those reported previously for (+)-torreyol and α-cadinol, respectively [31–33]. Taken together, we demonstrated that the sesquiterpene biosynthetic genes *GME3638* and *GME3634* are involved in the biosynthesis of (+)-torreyol and α-cadinol, respectively (Table 1; Fig. 1). Both compounds have been previously reported and identified as sesquiterpene alcohols isolated from plants but have been reported in only limited cases from fungi [31, 34–36].

### Phylogeny of STSs GME3634 and GME3638

**Fig. 3:**
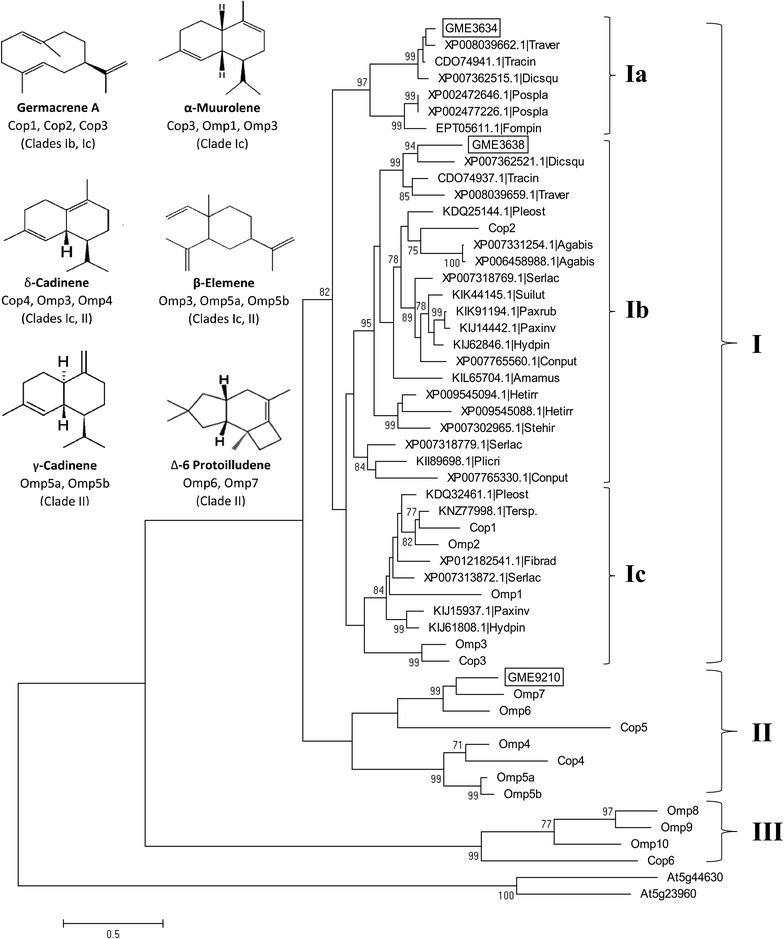
Phylogeny and distribution of GME3634, GME3638, and GME9210 from *L. rhinocerotis* TM02 with selected fungal sesquiterpene synthase homologs. Rooted maximum-likelihood tree with the highest log likelihood (−14290.4851) is shown. The *tree* is drawn to scale, with branch lengths measured in the number of substitutions per site. Percentage of trees in which the associated taxa clustered together is shown next to the *branches labelled* with gene names or gene accession numbers with abbreviated strain names

BLAST searches across fungal genomes showed that only a few of the STSs from basidiomycetes have been functionally characterised with the majority from recent genome-wide studies on *O. olearius* and *C. cinereus* [12, 14]. Phylogenetic analysis of GME3634, GME3638, and GME9210 against 50 other STS sequences (both putative and characterised) resulted in a phylogenetic tree revealing three distinct Basidiomycete sesquiterpene clades, termed Clades I, II, and III based on previous analysis [12, 14], with the outgroup consisting of the florally expressed terpene synthase genes At5g23960 and At5g44630 of *A. thaliana* [26] (Fig. 3). Both GME3634 and GME3638 (sharing 57.2% protein identity) were grouped into Clade I and were further segregated into subclade Ia and Ib, respectively. Ib consists of STSs from diverse basidiomycete fungi but Ia exclusively consists of uncharacterised STSs of the Polyporaceae family, including those from *T. versicolor*, *T. cinnabarina*, *Dichomitus squalens*, *Postia placenta*, and *Fomitopsis pinicola*, suggesting that STSs in subclade 1a are conserved taxonomically. Interestingly, the occurrence of GME3634 and GME3638 gene homologs as a pair in a gene cluster were also observed in *T. versicolor* (XP008039662.1/XP0080339659.1), *T. cinnabarina* (CDO74941.1/CDO74937.1), and *D. squalens* (XP007362515.1/XP007362521.1).

STSs from *O. olearius* and *C. cinereus* have been characterised in previous genome-wide studies using *E. coli* and/or *S. cerevisiae* as heterologous expression systems [12, 14]. The STSs from *C. cinereus* (Cop1—3) and *O. olearius* (Omp1—3) that utilize a 1,10-cyclization of (2*E*,6*E*)-farnesyl pyrophosphate (FPP) to produce sesquiterpenes derived from an (*E*,*E*)-germacradienyl cation fall into Clade I, but they formed separate subclades from GME3634 and GME3638. Cop1 and Cop2 synthesise germacrene A as the major product while Cop3 and Omp1 are α-muurolene synthases [12, 14]. In addition to α-muurolene, Omp3 also synthesises β-elemene, selina-4,7-diene, and δ-cadinene. Omp2 was reported to be not functional by Wawrzyn et al. [12]. In contrast to the Clade I STSs from *C. cinereus* and *O. olearius*, GME3634 and GME3638 produce the related sesquiterpene alcohols from 1,10-cyclization of (2*E*,6*E*)-FPP as major products, including germacrene-4-ol and cadinols (Fig. 1).

GME9210 was grouped into Clade II, which consists of Omp6 and Omp7 which is involved in the synthesis of the anticancer illudin precursor, Δ-6 protoilludene. Further pairwise sequence identity and similarity calculations from the multiple sequence alignment of GME9210 to Omp6 and Omp7 indicated that GME9210 is 58 and 62% identical and 64 and 68% similar to Omp6 and Omp7, respectively. Majority of STSs in this clade were postulated to share a common 1,11-cyclization of (2*E*,6*E*)-FPP mechanism, producing the trans-humulyl cation [12]. We were unable to obtain the sesquiterpene product from GME9210 in sufficient amount for NMR analysis due to the low yield in *S. cerevisiae*, though it was tentatively assigned with the putative structure 1,3,4,5,6,7-hexahydro-2,5,5-trimethyl-2H-2,4a-ethanonaphthalene (Table 1) based on GC–MS analysis. No biosynthetic pathway has been proposed for 1,3,4,5,6,7-hexahydro-2,5,5-trimethyl-2H-2,4a-ethanonaphthalene. It is yet unknown if the sesquiterpene could be formed via 1,11-cyclization or 1,6-cylization of FPP.

### (+)-Torreyol and α-cadinol exhibited selective cytotoxicity against MCF7 cells

**Fig. 4:**
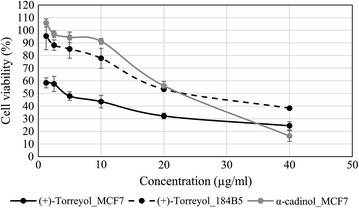
Cytotoxic activity of α-cadinol and (+)-torreyol in MCF7 and 184B5 cells. Effect of compounds on the viability of cells was examined by measuring relative cell viability via MTT assay (values are as mean ± SD, n = 3)

(+)-Torreyol and α-cadinol have been found to be a part of major constituents of essential oils of different plant species displaying cytotoxic effect against human oral, liver, lung and colon melanoma, and leukemic cancer cells [37, 38]. However, reports on the anticancer activity of these compounds in pure form are still limited. Thus, we examined the role of the isolated (+)-torreyol and α-cadinol as potential anticancer agent(s). We tested the anti-proliferative and cytotoxic activities of both compounds against MCF7 using an MTT assay. Both compounds showed marked toxicity against MCF7 cells where (+)-torreyol was approximately five times more potent than α-cadinol (Fig. 4). According to the criteria established by the USA National Cancer Institute (NCI), pure compounds with IC_50_ values less than 4 μg/ml following an incubation period of 48–72 h are considered to be cytotoxic agents [39]. Therefore, (+)-torreyol can be considered cytotoxic against MCF7 cells with IC_50_ of 3.5 ± 0.58 μg/ml. We further tested the cytotoxic effect of (+)-torreyol against A549, PC-3 and the non-tumorigenic 184B5 mammary gland cell line corresponding to MCF7. (+)-Torreyol showed less cytotoxicity to A549 (IC_50_ = 6.5 ± 3.28 µg/ml) and PC-3 cells (IC_50_ = 12.5 ± 1.50 µg/ml) while its cytotoxicity was at least sixfold lower towards 184B5 cells (IC_50_ = 22.7 ± 1.15 µg/ml) (Fig. 4). The selective cytotoxicity of (+)-torreyol against MCF7 makes it a good candidate for potential anticancer agent development.

## Discussion

*Saccharomyces cerevisiae* yeast has emerged as a powerful host cell for genome mining, biosynthetic studies, and production of fungal secondary metabolites, as it has many advantages [18, 19]. For expression of isoprenoid pathways, yeast readily provides the FPP substrate for STSs and has the added advantage of the ability to co-express functional cytochrome P450 monooxygenases that are often found encoded in terpene synthase gene clusters with compatible fungal cytochrome P450 reductase [40–43]. Successful expression of basidiomycete (Agaricales) STSs have been previously demonstrated in yeast [14]. However, this is the first report of the characterisation of STSs from the Polyporaceae family of mushrooms, which include several well-known medicinal mushrooms including *G. lucidum* and *Antrodia camphorata*.

Previous studies reported the expression of several sesquiterpene biosynthetic gene clusters in the genome and transcriptome of *L. rhinocerotis* [6, 7] but identification of their metabolic products remains elusive. In this study, we heterologously expressed three STSs namely GME3634, GME3638, and GME9210 in order to functionally characterise their metabolic products. GME9210 produced several sesquiterpenes at low abundances including 1,3,4,5,6,7-hexahydro-2,5,5-trimethyl-2H-2,4a-ethanonaphthalene (C_15_H_24_) while *GME3638* and *GME3634* encoded STSs that produce (+)-torreyol and α-cadinol, respectively, as major products. Both compounds are sesquiterpene alcohols and have been previously characterised but for the first time, the polypore *L. rhinocerotis* (Polyporaceae) has been shown to encode the pathways for biosynthesis of these sesquiterpene alcohols.

In this study, using the sequence and function data of the characterised STSs from *C. cinereus* (Cop1—6) and *O. olearius* (Omp1—10) as guide [12, 14], we extended the analysis by including the newly identified STSs from *L. rhinocerotis* as well as putative STS sequences from various Polyporaceae basidiomycetes to provide insights into the diversity and biochemical functions of STSs in *L. rhinocerotis.* It is interesting to note that GME3634 and GME3638 gene homologs exist as a pair in a gene cluster and are responsible for production of isomeric sesquiterpenes, (+)-torreyol and α-cadinol, with different stereochemistry. Similar paired GME3634/GME3638 homologs were also found in *T. versicolor*, *T. cinnabarina*, and *D. squalens* as clustered genes, suggesting that they are conserved in multiple Polyporaceae mushrooms. Their high homology and conserved synteny suggest that these STS homologs may be involved in the biosynthesis of (+)-torreyol and α-cadinol and/or related sesquiterpenes, which may serve common biological functions.

The occurrence of the major sesquiterpenic alcohols in the cultures of yeast harbouring *GME3634* and *GME3638* presents an interesting question whether these alcohols are the direct product of the enzymes. Wawrzyn et al. [12] suggested that (+)-torreyol from the Agaricales mushroom *O. olearius* is derived from α-muurolene synthesised by the α-muurolene synthase Omp1. A single P450 oxygenase gene collocated with *omp1* in the biosynthetic cluster was proposed to be responsible for converting α-muurolene to (+)-torreyol [12]. GME3638, which synthesises (+)-torreyol, shares 43% identity to Omp1. However, we have not detected the major production of α-muurolene in the culture of yeast expressing GME3638, but just a minor one. Instead, we have detected germacrene D-4-ol as the second major product in addition to (+)-torreyol (Fig. 1). The same is true for GME3634, in which the gene clustered with *GME3638* and produced the stereoisomer α-cadinol with germacrene D-4-ol as the second major product in yeast culture. Although we cannot completely ruled out that an endogenous yeast enzyme (e.g. a hydroxylase) could affect the end product of the two STSs observed in the yeast cultures, we proposed that GME3638 and GME3634 could produce (+)-torreyol and α-cadinol as the direct product resulting from hydrolysis of the muurolenyl or cadinenyl cation, respectively (Additional file 1: Figure S2). Further in vitro characterisation of the enzymes is required to elucidate the biosynthetic mechanism of GME3634 and GME3638.

Phylogenetic analysis indicates that GME3638 and GME3634 belong to the same clade (Clade I) together with the α-muurolene synthase Omp1, the germacrene A synthases Cop1 and Cop2 [14], as well as several putative uncharacterised STSs from closely related taxa (Fig. 3). Of note is that the germacranyl cation is the key intermediate of many cadinene group compounds (which includes muurolenes) [44, 45], while germacrene D and germacrene D-4-ol are constantly detected as a side product in the yeast strains expressing GME3634 and GME3638 (Table 1). This is congruent with the germacranyl cation being an essential biosynthetic intermediate of muurolenes and cadinenes (Additional file 1: Figure S2). In line with the previous study [12], it appears that although the STS phylogenetic analysis was unable to provide an accurate prediction of the structures of the sesquiterpene products at this stage due to the lack of characterised STSs from basidiomycetes, the grouping of the STSs into major clades gave a good indication of the initial cyclization step.

α-Cadinol is a common major constituent of various bioactive essential oils and plant extracts, such as those obtained from *Lindera nacusua*, *Zingiber nimmonii*, and *Beilschmiedia madang* [46–48]. A structure–activity relationship study of cadinane skeletal sesquiterpenes from Taiwania heartwood (*Taiwania cryptomerioides*) indicated that the presence of an equatorial hydroxyl (–OH) group at C-10 with a *trans*-fused configuration at the ring junction has a positive influence on their antifungal activities [36], such is the case of α-cadinol and therefore some antifungal activity may be expected. On the other hand, with a *cis*-fused rings feature, the torreyol belongs to the muurolene-type of compounds in contrast to the cadinene and their related cadinols which have a *trans*-fused ring structure [34]. Torreyol can be (+) or (−) depending if it is dextrorotatory or levorotatory; these isomers have been described by the names δ-cadinol, sesquigoyol, pilgerol, cedrelanol, and albicaulol [34]. The name torreyol is widely used due to its priority in usage. (+)-Torreyol was first isolated as white crystalline needles from the leaves of *Torreya nucifera* [49] and later in a few basidiomycetes including *Clitocybe illudens* (synonym *O. olearius*) grown on agar medium, *Lentinus lepideus*, and *Xylobolus frustulatus* [31, 50].

Investigations into elucidating the bioactive compounds in order to discover the scientific basis of the traditional uses of *L. rhinocerotis* recorded by ethnomycological surveys are currently emerging. In particular, its anticancer activities have been well explored by several research groups [9, 51, 52]. A partially purified fraction made up of two putative cytotoxic subtilisin-like fungal serine proteases has been recently reported to exhibit apoptosis-inducing activity in MCF7 cells [9] and more recently, Pushparajah et al. [52] synthesised a functional recombinant lectin-like fungal immunomodulatory protein using cDNA of *L. rhinocerotis*, which exhibit cytotoxicity against cancer cell lines. While the reported bioactive compounds are of high molecular weight protein molecules, here, we characterised three STSs from *L. rhinocerotis* using yeast heterologous system, which led to the discovery of (+)-torreyol that exhibited potent cytotoxicity against MCF7 and α-cadinol that is less active. To our knowledge, this is the first report on the bioactivity of (+)-torreyol and α-cadinol in pure form as these compounds are usually assayed in mixture. In view of the promising bioactivity of (+)-torreyol, future directions may include in-depth studies on its pharmacological activities in vivo and the underlying molecular mechanisms.

## Conclusions

Two cytotoxic sesquiterpene alcohols, (+)-torreyol and α-cadinol, were isolated and purified from the yeast system expressing the *L. rhinocerotis* STSs GME3638 and GME3634, respectively. (+)-Torreyol is selectively active against MCF7 cells with IC_50_ value of 3.5 ± 0.58 μg/ml. This study provided insights into the biocatalytic activities of basidiomycete STSs and provides a platform for further genome mining of bioactive secondary metabolites from medicinal mushrooms of the Polyporaceae family. Future work will focus on characterisation of other STSs in *L. rhinocerotis* as well as on elucidating the in vivo anticancer activity and mechanism(s) of action of (+)-torreyol.

## Additional file

**Additional file 1: Table S1.** Coding sequences of *GME3634*, *GME3638*, and *GME9210*. **Table S2.** PCR primers information. **Table S3.** 13C (150.903 MHz) and 1H (600.130 MHz) NMR spectral data for torreyol and α-cadinol (using CDCl_3_ as internal standard, 77.16 ppm). **Figure S1.** Mass spectrums of putative torreyol (1) and α-cadinol (2) to that of known compounds in NIST 05 library. **Figure S2.** Proposed general mechanism for the biosynthesis of torreyol and α-cadinol from farnesyl pyrophosphate.
