# Supplementary material for: Heterologous expression of cytotoxic sesquiterpenoids from the medicinal mushroom Lignosus rhinocerotis in yeast
Source: Microb Cell Fact. 2017 Jun 12;16:103. doi: 10.1186/s12934-017-0713-x (PMC5468996; doi:10.1186/s12934-017-0713-x)
Supplement: Supplementary file 1 — Additional file 1: Table S1. Coding sequences of GME3634, GME3638, and GME9210. Table S2. PCR primers information. Table S3. 13C (150.903 MHz) and 1H (600.130 MHz) NMR spectral data for torreyol and α-cadinol (using CDCl3 as internal standard, 77.16 ppm). Figure S1. Mass spectrums of putative torreyol (1) and α-cadinol (2) to that of known compounds in NIST 05 library. Figure S2. Proposed general mechanism for the biosynthesis of torreyol and α-cadinol from farnesyl pyrophosphate. [file 12934_2017_713_MOESM1_ESM.pdf]

# SUPPLEMENTARY INFORMATION

**Heterologous expression of cytotoxic sesquiterpenoids from the medicinal mushroom *Lignosus rhinocerotis* in yeast**

Hui-Yeng Yeannie Yap[1,3], Mariano Jordi Muria-Gonzalez[2†], Boon-Hong Kong[1], Keith A. Stubbs[3], Chon-Seng Tan[4], Szu-Ting Ng[4], Nget-Hong Tan[1], Peter S. Solomon[2], Shin-Yee Fung[1]*, and Yit-Heng Chooi[2,3]*

[1]Department of Molecular Medicine, Faculty of Medicine, University of Malaya, 50603 Kuala Lumpur, Malaysia; [2]Research School of Biology, The Australian National University, Canberra, Australian Capital Territory 2601, Australia; [3]School of Molecular Sciences, University of Western Australia, Crawley, Western Australia 6009, Australia; [4]Ligno Biotech, 43300 Balakong Jaya, Selangor, Malaysia

*Corresponding authors:

Y-H Chooi*, yitheng.chooi@uwa.edu.au; S-Y Fung*, syfung@um.edu.my

Co-authors' email addresses:

H-YY Yap, yean_ny_nie@yahoo.com; MJ Muria Gonzalez, jordi.muria@curtin.edu;

B-H Kong, bh_kong84@yahoo.com; KA Stubbs, keith.stubbs@uwa.edu.au;

C-S Tan, tanchonseng@gmail.com; S-T Ng, szuting@ligno.com.my;

N-H Tan, tanngethong@yahoo.com.sg; PS Solomon, peter.solomon@anu.edu.au;

Present address: †Centre for Crop and Disease Management, Curtin University, Perth, WA 6102, Australia.

**Table S1.** Coding sequences of *GME3634*, *GME3638*, and *GME9210*

| Gene ID | Coding sequence |
|---|---|
| *GME3634* | ATGGCTGTAACTCCTGCAAACGTCGCCTCCCCCGACTCGCAAGAGATCGTGCTCAAATTCCCCGATTTCATCTCTCCCATCCCCTACCCCCTTCG<br>CTGCCATGTGCAGGAGCGAGAAGTGTCACGCCAATCCGAGGAATGGCTCCTCAGCATGGCCAACTTCTCCGAGAAACAGCGCAGCAAGTTCCT<br>TACCCTCAACGGTGGTCTTCTTAGCGGCATGTGCTACATCGACTGCACGTTTGACGAACTGCGGGTCTGCACCGACTTCATGAACTTCCTGTTCA<br>CACTCGACGACTGGACGGACGAGTTCGACACGACCGGCACGCGGGGTCTCGCAGAGTGCGTCATGAACACGCTTTACTGGCCGCACAGCTACC<br>AGGCAGACACCGCCGCGCACCGCTTGACAAAGTCCTTCTGGGTCCGCATGAAGCAGACGGCAGGCCCGGGCTGTCAGCAGCGCCTCATGTCGA<br>CGCTCGACACGTACTTCCAGGCCATCATGCAACAGGCCGCCGACCGTGGCTCACATAACATTCCGGAGTTGGAGGAGTACATCTTGCTCCGCCG<br>AGACACGAGCGGGTGCAAGATCGGTTTCGCGTTCATCGAATACGCAGCCAACATTGATCTGCCCGACGACGTCATTGAGCACCCAATCATCAA<br>GGCCATGGCCGATGCGACCAATGATCTCGTTTCGTGGGCGAATGATGTCCTTTCGTACAACGCTGAACAGTCACGCGGAGACACGCACAACCTC<br>GTTTGTGTTCTCATGGCTCAGAACGGCCTCGACCGGCAGGGTGCGATCGAGCTGGCGGGCGAGCTATGGGAGAAGACCCTCCACTTGTTCTTCG<br>AGTGCCGCAAGAACGTCCCTTCGTGGGGCTCCGAGATCGACCGTGCCGTCGCTCTGTACATCCAGGGGCTCGAAGACTGGATTATCGCGAACG<br>CCGAGTGGAGCTTCGAGACGGAGCGCTACTTTGGCAAGGACGGGCATCTCGTGAAGAAGACGCGGCAGGTTACACTGCTTCCGGTCCGTACAG<br>CTGCG |
| *GME3638* | ATGCGCGCTCGATCGTTCATTCTTCCCGACCTCGTGTCGGATTGCCCATATACGCTCAGGTGCAACTCCAATTGCGAAGCTGTTGCGCGCGCTTC<br>AGAGGCCTGGATGCTCGAAGACGCGAATCTCTCACCGAAACGTCGTGACGCCTTCCTGCGTCTGCGGGGGGGAGAACTCACGGCAGCGTGCTA<br>CCCCGATACGGACGAGGCCTGCCTGCGAGTCGCCGCGGACTTCCTCAATTTCCTGTTTAGTTTGGACGACTGGTCGGACGAGTTCAGTATGGAG<br>GACACTTGCGGGCTCGCGCAGTGCGTCATGTGTGTGCTCCATGATCCTGATGACTTCCAGACTGAAAAGGCTGCTGGCAAGCTCGCCAAAAGCT<br>TTTTCAATCGGTTCCGGCAGACGGCGGGGCCGAGGTGTACTCGTCGATTCATCGATAGTATGGACCTCTTCTTTCATGCGATTGCACAGCAAGC<br>CCAGGACCGCGCGTCCGGGTCTGCTCCCTCACTCGAAGAATATGTGGCCCTCCGCGAGGACACGAGCGGGTGCAAGCCCTGCTTCGCTCTCATC<br>GAATACGCCGCGGGTATGGACCTTCCAGACCACGTCGCCCATCATCCAACAATCACCGCCCTCGAGCGGGAAGCCAACGCGTGTATATCATGG<br>TCGAACGATCTCTTCTCGTACAACGTCGAGCAAGCGCGGGGTGACACACACAACATGATTGCGGTGATCATGCGCGAGGACGGGCGCAGCTTG<br>CAAGAAGCCGTTGAATACTTGGGCGCTCTCTGCAAGCTCTGCATAGTGCACTTCGAGGAAAACCGAGCCATGCTGCCATCGTGGGGTCCGGAG<br>ATTGACGGGGAAGTCGACAGGTATGTGCTCGGCCTCCAGGACTGGATGGTCGGTGCCCTCCATTGGAGCTTCGACACCGCACGTTATTTCGGGG<br>ATGAAGGCCCTGCGATCAAGAAGCACGGTGTCGTGACACTACTACCGCGGAAGTCTTCATCG |
| *GME9210* | ATGCCTTTGTCTTCGTCTGTTGTCGCTTTCCGCCTTCCTGACACGCTTGGGTGCTGGCCATGGCGACGCTGCCTGAACACGCATTATGTGGAGGC<br>CAAACAGGATTCGGCATCCTGGCTGGAGTCATTCCACCCGTTCGGCCCCAAGGCACAAAGGGCCTTCAACAAGTGCGACTTCAACCTTCTCGCG<br>TCCCTAGCGTATCCGGTGGCCAGCAAAGACCAACTGCGTGCAGGGTGTGACCTGATGAACGTCTTCTTCGTCTTCGACGAGTACTCTGATGTCG<br>AGAACGAGAAGACGGTCCAGCAGCTCGCGGACATCATCATGGACGCACTGCGAAACCCTCACAAGCCTCGTCCTGCAGGAGAATCTCTCGTGG<br>GCGAAATCACTAGGCAGTTCTGGGCGCGCACGATCAAGGTAGCGAGCGAACCGTCGCAGCGCCGATTCATCGAGACCTTTGACGACTACTGCC<br>AGTCCGTAGTCCAACAGGCTGCGGACCGGTCGCAGAACCACTTGCGTGACGTCGAGAGCTACTTGGAGAACCGGAGGGAGAACATCGGCGCC<br>AAGCCGTCGTTTGCACTGCTCGAGTTGGACATGAACCTCCCCGACGAGGTGATCGAGCACCCGACCATCGTCAACCTGACGACATGGGCCATTG<br>ACATGATCATCCTGGGAAACGACATTGTCTCGTACAACGTGGAGCAGGCGCGTGGCGACGACGGACACAACGTCGTGACCATCGTCATGCATC<br>ACTACAACGTCGACGTGCAGGGTGCCATGGACCGCATCGCCGAGTGGCACCAGAGGCTGGCGGACCAGTTCCTCACCAACTACAACAAGCTGC<br>CGTCGTGGGGACGCGAGATTGACGCGCAAGTTGAGCGGTACATCCAGGGCATCGGAAACTGGGTACGCGCGAACGACGCATGGAGTTTCGAG<br>AGCGAGAGGTACTTTGGATTGAACGGACGCGAGATCGAGCAGAGTCGTTGGGTAACCCTACTCCCTCGGGTCTCCGCGGAGAAGCCTGCGGTT<br>GTG |

**Table S2.** PCR primers information

| Gene ID | Primer name | Sequence | Product size (bp) |
|---|---|---|---|
| *GME3634* | XW-GME3634-F | ATCAACTATCAACTATTAACTATATCGTAAT ACCAATGGCTGTAACTCCTGCAAAC | 410 |
| | GME3634-fg1-r | GCTTCATGCGGACCCAGAAGGACTTTGTCAA GCGGTGCG | |
| | GME3634-fg2-f | CGCACCGCTTGACAAAGTCCTTCTGGGTCCG CATGAAGC | 289 |
| | GME3634-fg2-SOE-r | GTCTCCGCGTGACTGTTCAGCGTTGTACGAA AGGACATCATTCGCCCACGAAACGAGATC | |
| | GME3634-fg3-SOE-f | GATGTCCTTTCGTACAACGCTGAACAGTCAC GCGGAGACACGCACAACCTCGTTTGTGT | 297 |
| | XW-GME3634-R | TGTCATTTAAATTAGTGATGGTGATGGTGAT GCACCGCAGCTGTACGGACCG | |
| *GME3638* | XW-GME3638-F | ATCAACTATCAACTATTAACTATATCGTAAT ACCAATGCGCGCTCGATCGTT | 374 |
| | GME3638-fg1-r | GCCGGAACCGATTGAAAAAGCTTTTGGCGAG CTTGCCAGC | |
| | GME3638-fg2-f | GCTGGCAAGCTCGCCAAAAGCTTTTTCAATC GGTTCCGGC | 289 |
| | GME3638-fg2-r | ACGTTGTACGAGAAGAGATC-GTTCGACCATGATATACACGCG | |
| | GME3638-fg3-f | CGTGTATATCATGGTCGAACGATCTCTTCTCG TACAACGTCGA | 336 |
| | XW-GME3638-R | TGTCATTTAAATTAGTGATGGTGATGGTGAT GCACCGATGAAGACTTCCGCGGTA | |
| *GME9210* | XW-GME9210-F | ATCAACTATCAACTATTAACTATATCGTAAT ACCAATGCCTTTGTCTTCGTCTGTTG | 178 |
| | GME9210-fg1-r | CTTTGCTGGCCACCGGATACGCTAGGGACGC GAGAAGGTTGAAGTCGCACTTGTTGAAGG | |
| | GME9210-fg2-f | ACCTTCTCGCGTCCCTAGCGTATCCGGTGGCC AGCAAAGACCAACTGCGTGCAGGGT | 177 |
| | GME9210-fg2-r | CTTGATCGTGCGCGCCCAGAACTGCCTAGTG ATTTCGCCCA | |
| | GME9210-fg3-f | GGGCGAAATCACTAGGCAGTTCTGGGCGCGC ACGATCAA | 282 |
| | GME9210-fg3-r | CACGTTGTACGAGACAATGTCGTTTCCCAGG ATGATCATGTCA | |
| | GME9210-fg4-f | CATGATCATCCTGGGAAACGACATTGTCTCG TACAACGTGGAG | 353 |
| | XW-GME9210-R | TGTCATTTAAATTAGTGATGGTGATGGTGAT GCACCACAACCGCAGGCTTCTCCG | |

**Table S3.** 13C (150.903 MHz) and 1H (600.130 MHz) NMR spectral data for torreyol and α-cadinol (using $CDCl_3$ as internal standard, 77.16 ppm)

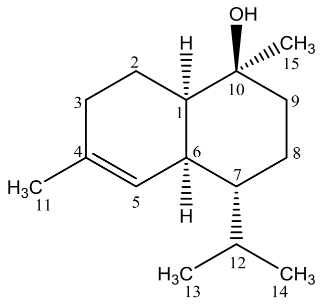


**1**

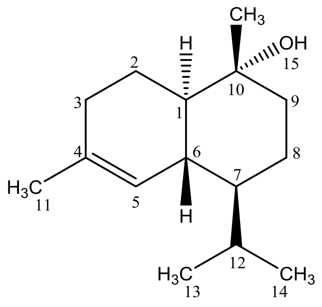


**2**

| **Carbon*** | **Torreyol (1)** | | **α-Cadinol (2)** | |
|---|---|---|---|---|
| | **δC (ppm)** | **δH (ppm)** | **δC (ppm)** | **δH (ppm)** |
| 1 | 45.55 d | 1.60 m | 50.15 d | 1.21 |
| 2 | 18.52 t | 1.89 m, 1.58 m | 22.09 t | 1.60, 1.14 |
| 3 | 31.14 t | 1.99 | 31.07 t | 1.98 |
| 4 | 134.37 s | --- | 135.12 s | --- |
| 5 | 124.61 d | 5.51 m | 122.46 d | 5.50 s |
| 6 | 36.79 d | 2.02 d | 40.00 d | 1.72 |
| 7 | 44.10 d | 1.3 | 46.83 d | 1.04 |
| 8 | 21.54 t | 1.51, 1.01 | 22.81 t | 1.27, 2.00 |
| 9 | 35.34 t | 1.56 t, 1.52 t | 42.33 t | 1.82, 1.80 |
| 10 | 72.58 s | --- | 72.62 s | --- |
| 11 | 23.64 q | 1.66 | 23.99 q | 1.67 s |
| 12 | 26.42 d | 1.97 m | 26.12 d | 2.15 m |
| 13 | 21.69 q | 0.89 d | 21.67 q | 0.92 d |
| 14 | 15.33 q | 0.81 d | 15.26 q | 0.77 d |
| 15 | 27.97 q | 1.29 | 20.91 q | 1.10 s |

*Carbon signal assignments are based on Van Eijk et al. [1].

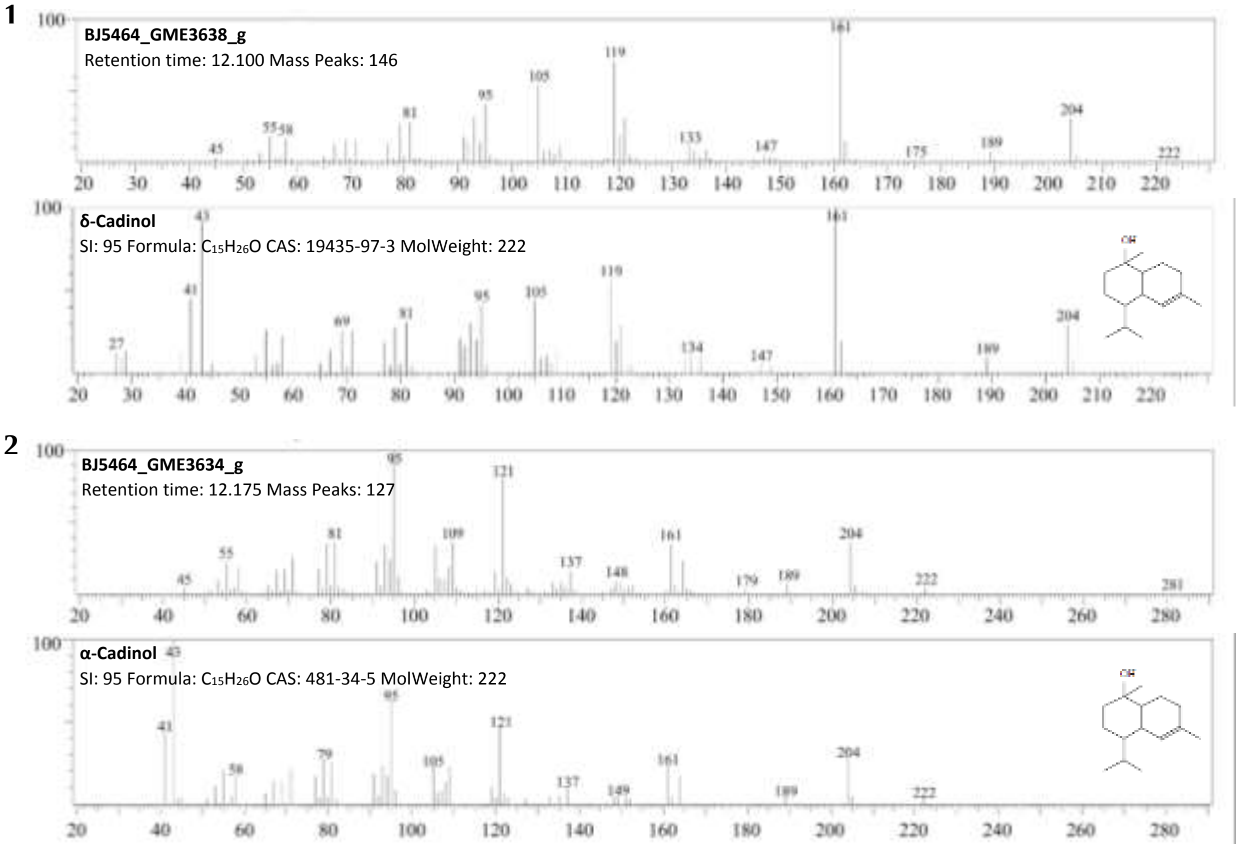


**Figure S1.** Mass spectrums of putative torreyol (1) and α-cadinol (2) to that of known compounds in NIST 05 library.

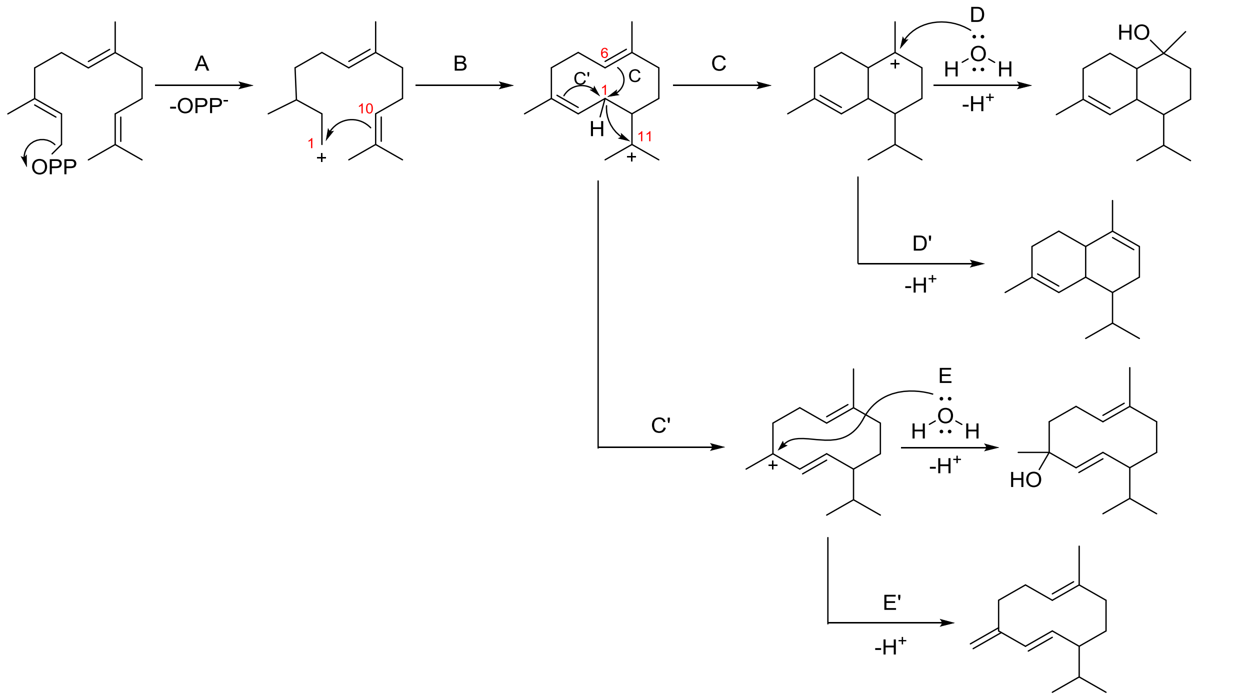


**Figure S2.** Proposed general mechanism for the biosynthesis of torreyol and α-cadinol from farnesyl pyrophosphate. The excision of the pyrophosphate group is catalysed by the enzyme producing the farnesyl cation (A: farnesyl carbocation formation). This is followed by the nucleophilic attack of carbon 10 (C-10) to the cation in C-1 that produces the first cyclisation reaction leading to germacranyl cation (B: cyclisation leading to germacranyl cation). At this point, the germacranyl cation can be quenched (C′: hydride shift) by deprotonation or hydrolysis to form germacrene (E: carbocation quenching by hydrolysis producing germacrene D-ol) and germacrene-4-ol [E’: hydride shift leading to a germacrene (germacrene D is presented here)] respectively, or continue the second cyclisation reactions via a Wagner-Meerwein rearrangement followed by the nucleophilic attack of C-6 over C-1. Depending on which face the hydride shift occurs, this leads to either the muurolenyl or cadinenyl cation (C: hydride shift). Finally, the resulted cation is quenched by either deprotonation [D’: hydride shift resulting in a cadinene (α-cadinene is shown here)] or hydrolysis (D: carbocation quenching by hydrolysis leading to torreyol and α-cadinol) to yield cadinene/muurolene or cadinols/muurolols, respectively. Note: The numbering in here follows the farnesyl numbering, not the final product numbering. In the case of GME3634 and GME3638, the deprotonation is a competing reaction that also yields sesquiterpene products that can be seen in Table 1; however, hydrolysis appears to be the preferred mechanism.

**Reference**


1. Van Eijk GW, Roeijmans HJ, Verwiel PEJ. Isolation and identification of the sesquiterpenoid (+)-torreyol from *Xylobolus frustulatus*. Exp Mycol. 1984;8:273-5.
